# A case of successful transarterial stenting for pseudoaneurysm after pancreaticoduodenectomy

**DOI:** 10.1002/jgh3.12345

**Published:** 2020-04-30

**Authors:** Fumimasa Kitamura, Hirohisa Okabe, Yo‐ichi Yamashita, Tatsunori Miyata, Hiromitsu Hayashi, Katsunori Imai, Akira Chikamoto, Hideo Baba

**Affiliations:** ^1^ Department of Gastroenterological Surgery Graduate School of Life Sciences, Kumamoto University Kumamoto City Japan

**Keywords:** post‐pancreaticoduodenectomy bleeding, Subtotal Stomach‐Preserving Pancreaticoduodenectomy (SSPPD), transarterial stenting

## Abstract

Postpancreaticoduodenectomy (PD) bleeding, which is reported to occur in 5–7%, is a major complication that often causes life‐threatening secondary events. A transarterial catheter technique with coil embolization is a widespread procedure that could potentially cause massive hepatic infarction and subsequent sepsis with hepatic abscess, which can be a fatal complication. Here, we introduce a new transarterial technique that uses a hemostat with a stent graft, which successfully rescued a patient had suffered post‐PD bleeding.

## Graphical abstract

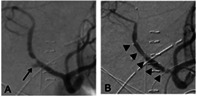

This case demonstrated the successful treatment of a patient with post‐PD bleeding by transarterial stenting. Although sufficient experience and skill in angiographic techniques are required of is mandatory, and the need for a stent is largely determined by the arterial anatomy, we suggest that this technique may be promising for improving short‐term outcomes for post‐PD bleeding.

## Introduction

Postpancreaticoduodenectomy (PD) bleeding, which is reported to occur in 5–7%, is a major complication that often causes life‐threatening secondary events. A transarterial catheter technique with coil embolization is a widespread procedure that could potentially cause massive hepatic infarction and subsequent sepsis with hepatic abscess, which can be a fatal complication. Here, we introduce a new transarterial technique that uses a hemostat with a stent graft, which successfully cured a patient suffering from post‐PD bleeding.

## Case report

**Figure 1:**
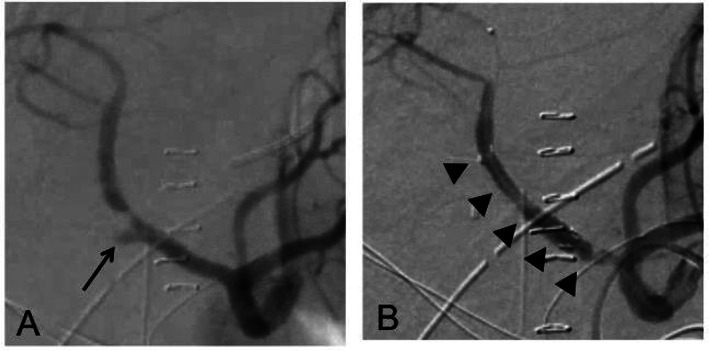
Digital subtraction angiography at sentinel bleeding and stenting for the gastroduodenal artery. (a) A small aneurysm was noted at GDA stump (arrow). (b) A stent graft, 5 mm in diameter and 2.5 cm in length, was placed in the CHA (arrowheads). CHA, common hepatic artery; GDA, gastroduodenal artery.

**Figure 2:**
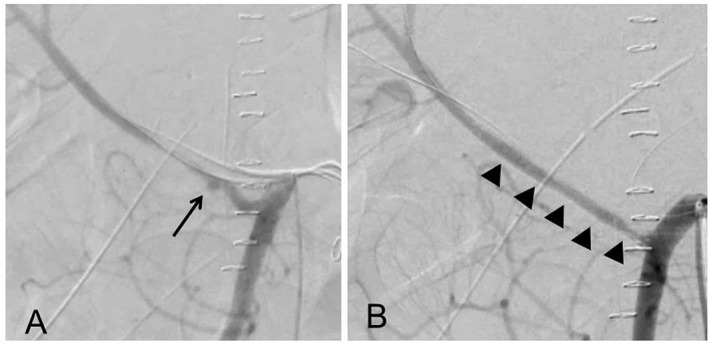
Digital subtraction angiography at sentinel bleeding and stenting for the right hepatic artery replaced on the superior mesenteric artery. (a) A small aneurysm was noted at RHA replaced on SMA (arrow). (b) A stent graft, 5 mm in diameter and 5 cm in length, was placed in the RHA (arrowheads). RHA, right hepatic artery; SMA, superior mesenteric artery.

A 68‐year‐old male patient was referred to our hospital with distal bile duct carcinoma and underwent PD. Intraoperative findings were that the pancreas was soft and the diameter of the pancreatic duct was less than 2 mm. A pancreatic fistula was noted, and C‐reactive protein (CRP) level reached 39 mg/dL at postoperative day 3. Although body temperature did not exceed 37.5°C, and CRP level gradually decreased to 3 mg/dL, sentinel bleeding occurred at postoperative day 12, with vital signs remaining stable. An emergency angiographic examination was performed. It was found that there might be two small aneurysms: one on the stump of the gastroduodenal artery (Fig. 1a) and another on the stump of the inferior pancreaticoduodenal artery (IPDA) branching from the right hepatic artery (RHA) replaced on the superior mesenteric artery (SMA) (Fig. 2a). Both aneurysms were suspected to be responsible for the sentinel bleeding, although no extravasation was evident during the angiographic examination. A stent graft was placed in both the common hepatic artery (CHA), isolating the stump of the GDA (Fig. 1b), and the replaced RHA (Fig. 2b), isolating the stump of the IPDA to prevent massive rupture of the aneurysms. The hepatic flow of the LHA and RHA was preserved. The intraperitoneal abscess adjacent to both arteries was subsequently treated by appropriately changing the intraperitoneal drains around the pancreaticojejunostomy. The patient recovered and was discharged on postoperative day 45.

## Discussion

The effectiveness of endovascular treatment with stenting for delayed post‐PD bleeding has been reported. This is a very rare case of a patient who showed two pseudoaneurysms on both the GDA stump and the IPDA stump on the replaced RHA, successfully treated by VIABAHN® stent grafts preserving the intraluminal blood flow. The size of the stent should be considered depending on the location of bleeding. In the current case, the RHA was replaced on the SMA, and the size of RHA was relatively small for the 5 mm‐diameter stent, which is the smallest available stent size. We had planned to treat the replaced RHA with coil embolization in case the diameter of the artery was too small to allow the stent to be inserted. Eventually, the aneurysm on the IPDA stump on the RHA was successfully repaired with the stent, and the aneurysm on the GDA was also treated with a 5 mm‐diameter stent.

The technique used in the current case may aid in the improvement of mortality after PD. Angiographic treatment is less invasive than surgical treatment, and with appropriate use of a hemostat to maintain the blood flow, the concerns of hepatic infarction and subsequent sepsis with hepatic abscess after the embolization of the hepatic artery were alleviated.

In conclusion, this case demonstrated the successful treatment of a patient with post‐PD bleeding by transarterial stenting. Although sufficient experience and skill in angiographic techniques are required, and the need for a stent is largely determined by the arterial anatomy, we suggest that this technique may be promising for improving short‐term outcomes for post‐PD bleeding.

